# Should prenatal care providers offer pregnancy options counseling?

**DOI:** 10.1186/s12884-018-2012-x

**Published:** 2018-09-27

**Authors:** Nancy F. Berglas, Valerie Williams, Katrina Mark, Sarah C. M. Roberts

**Affiliations:** 1 Advancing New Standards in Reproductive Health, Department of Obstetrics, Gynecology and Reproductive Sciences, University of California, San Francisco, 1330 Broadway, Suite 1100, Oakland, CA 94612 USA; 2 Department of Obstetrics and Gynecology, Louisiana State University Health Sciences Center New Orleans, 1542 Tulane Avenue, Box T5-2, New Orleans, LA 70112 USA; 3 Department of Obstetrics, Gynecology and Reproductive Sciences, University of Maryland School of Medicine, 655 W. Baltimore Street, Baltimore, MD 21201 USA

**Keywords:** Abortion, Adoption, Pregnancy options counseling, Prenatal care, Screening

## Abstract

**Background:**

Professional guidelines indicate that pregnancy options counseling should be offered to pregnant women, in particular those experiencing an unintended pregnancy. However, research on whether pregnancy options counseling would benefit women as they enter prenatal care is limited. This study examines which women might benefit from options counseling during early prenatal care and whether women are interested in receiving counseling from their prenatal care provider.

**Methods:**

At four prenatal care facilities in Louisiana and Maryland, women entering prenatal care completed a self-administered survey and brief structured interview (*N* = 586). Data were analyzed through descriptive statistics, bivariate analyses, multivariate multinomial logistic regression, and coding of open-ended responses.

**Results:**

At entry into prenatal care, most women reported that they planned to continue their pregnancy and raise the child. A subset (3%) scored as having low certainty about their decision on the validated Decision Conflict Scale, indicating need for counseling. In addition, 9% of women stated that they would be interested in discussing their pregnancy options with their prenatal care provider. Regression analyses indicated some sociodemographic differences among women who are in need of or interested in options counseling. Notably, women who reported food insecurity in the prior year were found to be significantly more likely to be in need of options counseling (RRR = 3.20, *p* < 0.001) and interested in options counseling (RRR = 5.48, p < 0.001) than those who were food secure. Most women were open to discussing with their provider if their pregnancy was planned (88%) or if they had considered abortion (81%). More than 95% stated they would be honest with their provider if asked about these topics.

**Conclusions:**

Most women are certain of their decision to continue their pregnancy at the initiation of prenatal care. However, there is a subset of women who, despite entering prenatal care, are uncertain of their decision and wish to discuss their options with their health care provider. Screening tools and/or probing questions are needed to support prenatal care providers in identifying these women and ensuring unbiased, non-directive counseling on all pregnancy options.

## Background

Health care providers play an important role in ensuring that women have the information and services they need to make informed decisions about their pregnancies. Pregnancy options counseling is an opportunity for the pregnant woman to consider, in conversation with her provider, whether she wants to continue the pregnancy and parent the child, continue the pregnancy with a plan for adoption, or have an abortion. Nearly half of pregnancies in the U.S. are unintended, [1] indicating there may be a substantial number of women who might benefit from options counseling in early pregnancy.

A number of leading professional societies have produced guidelines on offering information and referrals for prenatal care, adoption services, and abortion services to pregnant patients [2–5]. These guidelines describe a provider’s professional and ethical obligation to provide unbiased, non-directive counseling on all available pregnancy options or, if he or she cannot due to personal beliefs, make a timely referral to another provider. This obligation is framed as fundamental to respecting patients’ autonomy.

Patient-centered care—respectful and responsive to individual patient preferences, needs and values [6]—requires understanding of patient experiences, and yet there is little research that elicits women’s own views on pregnancy options counseling. In one qualitative study with 28 women at prenatal and abortion clinics in Nebraska, most women voiced support for offering options counseling to all pregnant women. [7] They expressed the need for comprehensive, unbiased information given with respect for their autonomy, free from assumptions about their preferred outcome for the pregnancy, and tailored to their medical and social circumstances.

Assessing women’s certainty about her decision to have an abortion has become a regular component of abortion care [8]. However, the availability and timing of pregnancy options counseling beyond the context of abortion is not routine and has not been widely explored. It is not clear whether counseling should be provided to all women, directed at some women, or given only if requested. It is also unclear at what point in care that options counseling should be provided. The pregnancy test visit may be an opportune time to open this discussion, [9] but many women determine that they are pregnant outside of the health care system using home pregnancy tests. Many choose not to address an unintended pregnancy with their regular gynecologist before seeking abortion [10]. Furthermore, the availability of counseling varies by provider and setting. Many primary care providers do not address all pregnancy options as part of routine care, even when they support the availability of counseling in concept [11]. For women who receive pregnancy tests at publicly-funded family planning clinics, the availability of options counseling varies by type of facility [12].

For all these reasons, the initial visit to a prenatal care provider may be a woman’s first opportunity to speak with a provider about her pregnancy options. The first prenatal visit tends to be longer than subsequent visits, offering time for in-depth conversation that allows the provider to understand a woman’s individual circumstances, the context of her pregnancy, and her need for additional support and resources [13]. However, through training and at the guidance of their professional societies, providers are tasked with completing many required practices as part of prenatal care, including medical screening and treatment, promotion of positive health behaviors, and psychosocial support [14]. Time is limited, and it is important for providers to be able to prioritize care based on scientific evidence in concert with individual patient needs. To that end, it needs to be determined whether universal pregnancy options counseling is a necessary and beneficial part of early prenatal care.

In this exploratory study, we aim to understand which pregnant women, if any, might benefit from pregnancy options counseling upon entry into prenatal care. We base our determination on a validated measure of decision certainty about the pregnancy (as an objective indication of clinical need), as well as a direct question of interest in receiving counseling from their provider (as an indication of patient preference). We also seek to understand women’s level of comfort discussing their pregnancy intentions and options with their provider, as an indication of potential harm should providers raise the topic of pregnancy options during a visit.

## Methods

### Study design and setting

The current analysis is part of a large cross-sectional study, the Multistate Abortion Prenatal Study (Roberts SCM, Kimport K, Kriz R, Holl J, Mark K, Williams V: Consideration of and reasons for not obtaining abortion among women entering prenatal care in Southern Louisiana and Baltimore, Maryland, forthcoming). We recruited English- and Spanish-speaking women ages 18 and older who presented for their first prenatal visit at four prenatal care facilities in Southern Louisiana and Baltimore, Maryland between June 2015 and July 2017. These locations were selected for their similar demographic profiles in terms of race/ethnicity, poverty and birth rate, but different reproductive health policy environments. The prenatal care facilities were affiliated with local universities and served primarily low-income pregnant women, many of whom were eligible for Medicaid for their prenatal care. The institutional review boards of the University of California, San Francisco (UCSF) and Louisiana State University approved the study protocol. The University of Maryland’s institutional review board relied on the approval of the UCSF institutional review board.

### Participants

At each facility, an onsite research coordinator approached eligible women and invited them to participate in the study. Women were eligible if they were at least 18 years old, were pregnant, were presenting for their first prenatal care visit, and spoke and read English or Spanish. Women who were younger than 18, were not pregnant, were there for a subsequent prenatal care visit, did not speak and read English or Spanish, or were incarcerated were ineligible. After participants provided written consent, the research coordinator demonstrated how to complete a self-administered iPad survey and left them to complete it independently. The research coordinator then conducted a brief (5 to 15 min) structured interview in a private space. Women received a $30 gift card for their participation.

### Measures

On the self-administered survey, participants were asked their current preferred outcome for this pregnancy: having the baby and raising it, adoption or having someone else raise it, or abortion. Participants were also asked on both the self-administered survey and during the in-clinic interview if they had considered abortion at any point (“for just one second”) during their pregnancy. We considered a participant as having considered abortion if they responded affirmatively either on the survey or during the interview.

Participants completed the Decisional Conflict Scale (DCS) based on their preferred pregnancy outcome. The DCS is a validated scale used to assess individuals’ perceptions of certainty in the context of health care decisions [15]. The DCS includes 16 items, answered on a 5-point Likert scale to indicate level of agreement (strongly disagree to strongly agree). Scores are transformed to range from 0 to 100, with lower scores reflecting lower levels of conflict and greater certainty about a decision. Scores greater than 37.5 have been found to be associated with delay and difficulty implementing a decision, and are considered to be of clinical concern [16]. These were categorized dichotomously as “low decision certainty.” We considered these women as being *in need of pregnancy options counseling*.

During the interview, participants were asked whether they would like to discuss their pregnancy options with a doctor or nurse. Participants could indicate if they wanted counseling, did not want counseling, and/or had already received counseling during this pregnancy. Although participants could provide more than one response, the categories were mutually exclusive in this sample, resulting in a categorical variable. We considered women who responded that they “wanted counseling” as *interested in pregnancy options counseling*.

Participants were asked whether their provider could ask them about sensitive health topics, including whether this pregnancy was planned and if they had considered abortion. Participants were also asked how honest they would be if their provider asked whether their pregnancy was planned and if they had considered abortion. Participants answered each item on a 5-point Likert scale. Women who disagreed or strongly disagreed were asked open-ended questions about the reasons they might not want to talk to their provider about these topics.

Other variables on the self-administered survey included age, race/ethnicity, highest level of education, employment status, use of public assistance in the last 12 months, food insecurity in the last 12 months, gestational age of the pregnancy, trimester entering prenatal care, pregnancy intentions (measured using the London Measure of Unplanned Pregnancy (LMUP) [17]), and pregnancy history.

### Analysis

Data were analyzed through descriptive statistics, χ^2^ and t-tests, multivariate logistic regression, and coding of open-ended responses using Stata version 15 software (College Station, TX). To examine predictors of need for or interest in options counseling, we first assessed bivariate relationships with patient characteristics using χ^2^ tests for categorical variables and t-tests for continuous variables. We used multivariate multinomial logistic regression with a three-category outcome variable (needing options counseling, interested in options counseling, or not needing or interested in options counseling) to understand whether the predictors differed for each group. Four women were both in need of and interested in options counseling; they were categorized as interested in counseling in the model. A sensitivity analysis, categorizing these four women as in need of counseling, yielded similar patterns of results and are not reported. We adjusted for participant characteristics that were significant in bivariate analyses and used clustered standard errors (using Stata’s vce cluster command) to account for non-independent observations within recruitment facility. We calculated predictive probabilities based on the regression model (using Stata’s margins command).

## Results

### Sample description

Onsite research coordinators approached 753 women at the four prenatal care facilities; 91% (*n* = 685) were found to be eligible for the study. Of those eligible, 86% (*n* = 589) consented to participate. The final sample included 586 women who initiated the self-administered survey and in-clinic interview.

The mean age of participants was 27 years, ranging from 18 to 44 (Table 1). Most participants were African American (79%), had completed high school (80%), and had received public assistance in the past year (75%). About half reported being unemployed (49%) and having food insecurity in the past year (47%). Most had been pregnant before (80%), and about one-quarter had previously had an abortion (28%). Most (72%) were in the first trimester of pregnancy upon entry into prenatal care.

**Table 1:** Description of sample of pregnant women at first prenatal visit (*N* = 586)

Variable	n (%) or mean ± SD
Age, in years (M, SD)	27.0 **±** 5.6
Race/ethnicity	
White	45 (7.7)
Black or African American	461 (78.8)
Hispanic/Latina	55 (9.4)
Other/Multiple	24 (4.1)
Highest level of education	
Less than high school	120 (20.5)
Completed high school or GED	286 (48.9)
Some or completed college	179 (30.6)
Employment	
Employed full time	176 (30.2)
Employed part time	122 (20.9)
Unemployed	285 (48.9)
Public assistance in last 12 months	431 (75.5)
Food insecurity in last 12 months	271 (46.9)
Prior pregnancy	467 (80.1)
Prior birth	401 (68.6)
Prior abortion	165 (28.3)
Trimester entered prenatal care	
1st trimester	417 (72.2)
2nd trimester	130 (22.5)
3rd trimester	31 (5.4)
Gestational age of pregnancy, in weeks (M, SD)	11.3 **±** 8.1
London Measure of Unplanned Pregnancy score (M, SD)	7.0 **±** 2.9

### Preferred pregnancy outcome

Nearly all women (97%, *n* = 564) reported their preferred pregnancy outcome as having and raising the child. Two percent of women (*n* = 10) reported preferring for the child to be adopted, and 1% (*n* = 8) reported preferring to have an abortion. Among the eight women who currently preferred abortion, all were in their first trimester of pregnancy, within the gestational limit for abortion in their state. Among all women in the sample, nearly one-third (31%, *n* = 182) reported considering abortion at some time during this pregnancy.

### Decision certainty (needing options counseling)

Overall, DCS scores were low (mean 10.3, median 3.1), indicating high decision certainty among women at the first prenatal care visit. Mean DCS scores were significantly higher (indicating lower certainty) for women currently preferring adoption or abortion, compared to woman preferring to raise the child (27.2 adoption, 23.9 abortion, 9.9 raise child, *p* < 0.001). Twenty women (3%) were categorized as having low decision certainty (DCS > 37.5), indicating need for pregnancy options counseling.

In bivariate analyses, low decision certainty was significantly associated with participants’ state of residence, race/ethnicity, education, food insecurity, and LMUP score.

### Interest in options counseling

Most women (88%, *n* = 499) reported that they were not interested in discussing their pregnancy options with their provider at their prenatal care visit. Nine percent (n = 49) stated that they would like to discuss their pregnancy options, and 3% (*n* = 20) reported that they had already discussed their options with a provider. Women who currently preferred adoption or abortion were significantly more likely to be interested in discussing their pregnancy options with their provider, compared to women preferring to raise the child (30% adoption, 38% abortion, 8% raise child, *p* < 0.001).

In bivariate analyses, interest in pregnancy options counseling was significantly associated with participants’ state of residence, age, race/ethnicity, food insecurity, and pregnancy history.

### Need for vs. interest in pregnancy options counseling

There was little overlap between the 20 women who reported low decision certainty and 49 women who expressed interest in options counseling. Only four women were categorized as both in need of and interested in options counseling.

### Predictors of needing or being interested in pregnancy options counseling

The results of the multivariate regression model are presented in Table 2; the base outcome for the model is women who reported neither needing nor being interested in counseling. Compared to these women, women interested in options counseling were more likely to be living in Louisiana (RRR = 4.95, *p* < 0.001) and Hispanic/Latina (RRR = 3.00, *p* < 0.05). As might be predicted, higher LMUP scores – indicating more planned pregnancies – were associated with less need for (RRR = 0.77, *p* < 0.01) or interest in (RRR = 0.90, *p* < 0.001) options counseling.

**Table 2:** Multivariate multinomial logistic regression model predicting in need of or wanting pregnancy options counseling among pregnant women at their first prenatal visit (*n* = 570)

	Relative Risk Ratio	*p*-value	95% Confidence Interval	
**In need of options counseling**				
State				
Maryland	Reference			
Louisiana	1.29	n.s.	0.97	1.72
Age (cont.)	0.98	n.s.	0.94	1.02
Race/Ethnicity				
Black or African American	Reference			
White	1.02	n.s.	0.10	10.14
Hispanic/Latina	3.41	n.s.	0.32	36.30
Other/Multiple	2.01	n.s.	0.15	26.14
Education				
Did not complete high school	Reference			
High school or GED	0.38	*	0.18	0.82
Some or completed college	0.17	n.s.	0.01	2.11
Food insecurity in past 12 months	3.20	***	2.50	4.08
Previous abortion	0.83	n.s.	0.38	1.83
Previous pregnancy	0.41	n.s.	0.09	1.87
Gestational age (cont.)	1.01	n.s.	0.97	1.05
LMUP score (cont.)	0.77	**	0.64	0.91
**Wanting options counseling**				
State				
Maryland	Reference			
Louisiana	4.95	***	3.50	7.00
Age (cont.)	0.94	*	0.88	0.99
Race/Ethnicity				
Black or African American	Reference			
White	1.05	n.s.	0.43	2.57
Hispanic/Latina	3.00	*	1.10	8.22
Other/Multiple	0.75	n.s.	0.13	4.36
Education				
Did not complete high school	Reference			
High school or GED	1.45	n.s.	0.93	2.28
Some or completed college	1.22	n.s.	0.45	3.30
Food insecurity in past 12 months	5.48	***	2.49	12.03
Previous abortion	0.37	*	0.16	0.88
Previous pregnancy	0.76	n.s.	0.24	2.40
Gestational age (cont.)	1.00	n.s.	0.97	1.03
LMUP score (cont.)	0.90	***	0.86	0.95

*p < 0.05, **p < 0.01, ***p < 0.001, n.s. = not significant

Base outcome: Women not in need of or wanting pregnancy options counseling. Four women reporting both in need of and wanting counseling were categorized in model as wanting pregnancy options counseling. LMUP = London Measure of Unplanned Pregnancy

**Fig. 1:**
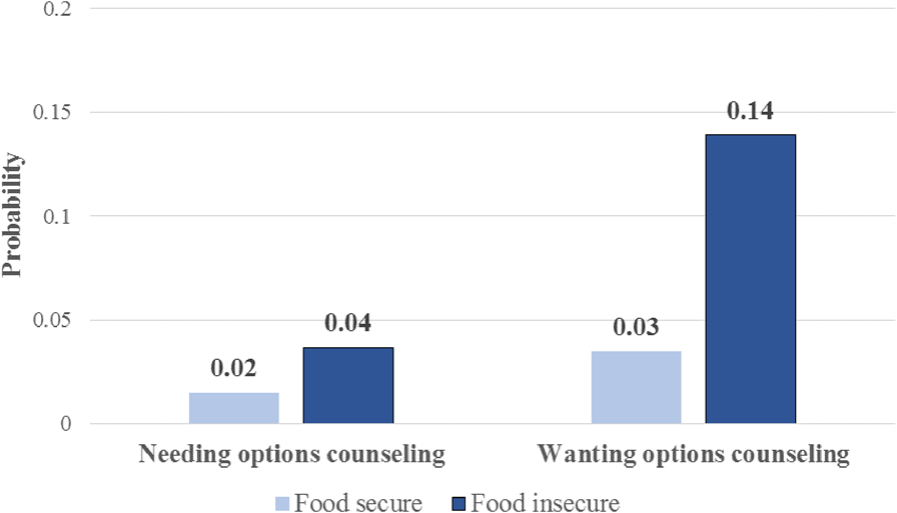
Predictive margins of needing or wanting pregnancy options counseling, by food insecurity status, based on multivariate multinomial logistic regression model (*n* = 570)

Notably, the model indicates significant differences by food insecurity. Women who reported food insecurity in the prior year were three times more likely to be in need of options counseling (RRR = 3.20, p < 0.001) and five times more likely to be interested in options counseling (RRR = 5.48, p < 0.001) than those who had not experienced food insecurity. The predicted probabilities of needing or being interested in pregnancy options counseling by food insecurity status are presented in Fig. 1.

### Provider questions about pregnancy intentions and abortion

Most women agreed that their providers can ask if their pregnancy was planned (88% agree/strongly agree, *n* = 498) and whether they had considered having an abortion during their pregnancy (81% agree/strongly agree, *n* = 462).

Among women who stated that providers should not ask if their pregnancy was planned (5% disagree/strongly disagree, *n* = 31), all planned to raise the child. Among women who stated that providers should not ask whether they had considered abortion (9% disagree/strongly disagree, *n* = 66), 95% planned to raise the child. Among the 20 women with low decision certainty, most were comfortable with their provider asking if their pregnancy was planned (*n* = 19) or if they had considered abortion (*n* = 17).

Nearly all women reported that they would answer honestly if their providers asked if their pregnancy was planned (97% agree/strongly agree, *n* = 552) and whether they had considered having an abortion (95% agree/strongly agree, *n* = 539).

## Discussion

Upon entry into prenatal care, most women felt certain about their decision to continue their pregnancy and raise the child, and most indicated that they were not interested in discussing their pregnancy options at this time. However, a not-insignificant minority of women felt less sure about their pregnancy. Women who preferred adoption had particularly high rates of low decision certainty. A few women preferred to terminate their pregnancy but presented for prenatal care despite being within the gestational limit for abortion in their state of residence. This indicates that the mere act of presenting to a prenatal care visit does not necessarily imply that the woman is certain of her plan to continue the pregnancy. Moreover, 9% of women expressed interest in options counseling at their first prenatal visit, even if they were not considered clinically “in need.”

Our analyses indicate that women in need of and/or interested in pregnancy options counseling differ from other pregnant women entering prenatal care. Not surprisingly, their pregnancies were less likely to be intended. Those who requested options counseling were more likely to be younger, Latina, and living in Louisiana – where women may face more policy barriers to accessing abortion. Most notably, food insecurity with the past year was highlighted as a significant predictor of both need for and interest in options counseling. Even within a relatively low-income population of women, those experiencing immediate economic insecurities were in greater need of support from their provider. Their needs are clearly material, but their interest in discussing their pregnancy options indicates that they are looking to providers for less tangible support as well.

It is worth noting that these interviews were conducted at women’s first prenatal care visits and not at the time that they obtained a positive pregnancy test. As many women take home pregnancy tests, it is possible that presenting to a prenatal care provider is the only way that they are familiar with to engage in the health care system and/or discuss options for their pregnancy. It stands to reason that women would be most comfortable speaking to their provider about their pregnancy options, just as is expected with other medical decision-making. If their provider assumes that their engagement in prenatal care implies their intent to continue the pregnancy, an opportunity may be missed to provide women with the counseling and autonomy that they need to fully understand and consider all of their options.

This study’s findings about food insecurity make clear how much these broader circumstances play a role in pregnancy decision-making and outcomes. Women who were food insecure were much more likely to be uncertain of their decision to continue pregnancy and be interested in pregnancy options counseling. This elucidates the fact that pregnancy options counseling does not consist solely of explaining the three seemingly obvious options (raising a child, adoption, or abortion), but that it should also include a more comprehensive discussion of how to best provide the support a woman needs to attain her desired pregnancy outcome. The question remains how to identify those women who are most in need of support, and then how to ensure that they are connected with necessary services. Screening tools and/or probing questions are needed to support prenatal care providers in identifying these women and ensuring unbiased, non-directive counseling on all pregnancy options.

This study has limitations. First, women’s reports about their preferences for abortion or adoption may be underreported due to stigma [18, 19]. We note, however, that more than 30% of women disclosed they had considered abortion during this pregnancy, indicating that our study procedures did encourage women to disclose. Nonetheless, to the extent that women may be unwilling to disclose their preference for their pregnancy, we may not fully understand which women might benefit from options counseling at the first prenatal visit. Second, we did not recruit participants younger than age 18; thus, the results may not represent the need for options counseling among pregnant minors. The American Academy of Pediatrics’ guidelines on options counseling may prove useful to providers working with younger patients [5]. Third, the women in this sample are primarily African American, low-income, and living in more urban settings, and all were recruited at large prenatal clinics. Although women were recruited from all levels of prenatal care, including midwifery, low-risk obstetrics and high-risk obstetrics, their experiences and needs may not be representative of all women entering prenatal care. The current body of research on the value of including pregnancy options counseling in early prenatal care is limited to this study and one other [7]. Both quantitative and qualitative research are needed to understand the value and potential impact of options counseling for different populations of women living in varied contexts.

## Conclusions

Our study finds that most women are certain of their decision to continue their pregnancy at the initiation of prenatal care. These results mirror research indicating that most women are certain of their decision when they present for an abortion [20–22]. In both settings, the options for the pregnancy are generally well known and women’s decisions about whether to proceed with an unintended pregnancy are typically made prior to reaching the health care system. However, there is a subset of women who, despite entering prenatal care, are uncertain of their decision and wish to discuss their options with their provider.
